# Supplementary figures and images for: Dynamic Transcript Profiling of Candida albicans Infection in Zebrafish: A Pathogen-Host Interaction Study
Source: PLoS One. 2013 Sep 3;8(9):e72483. doi: 10.1371/journal.pone.0072483 (PMC3760836; doi:10.1371/journal.pone.0072483)

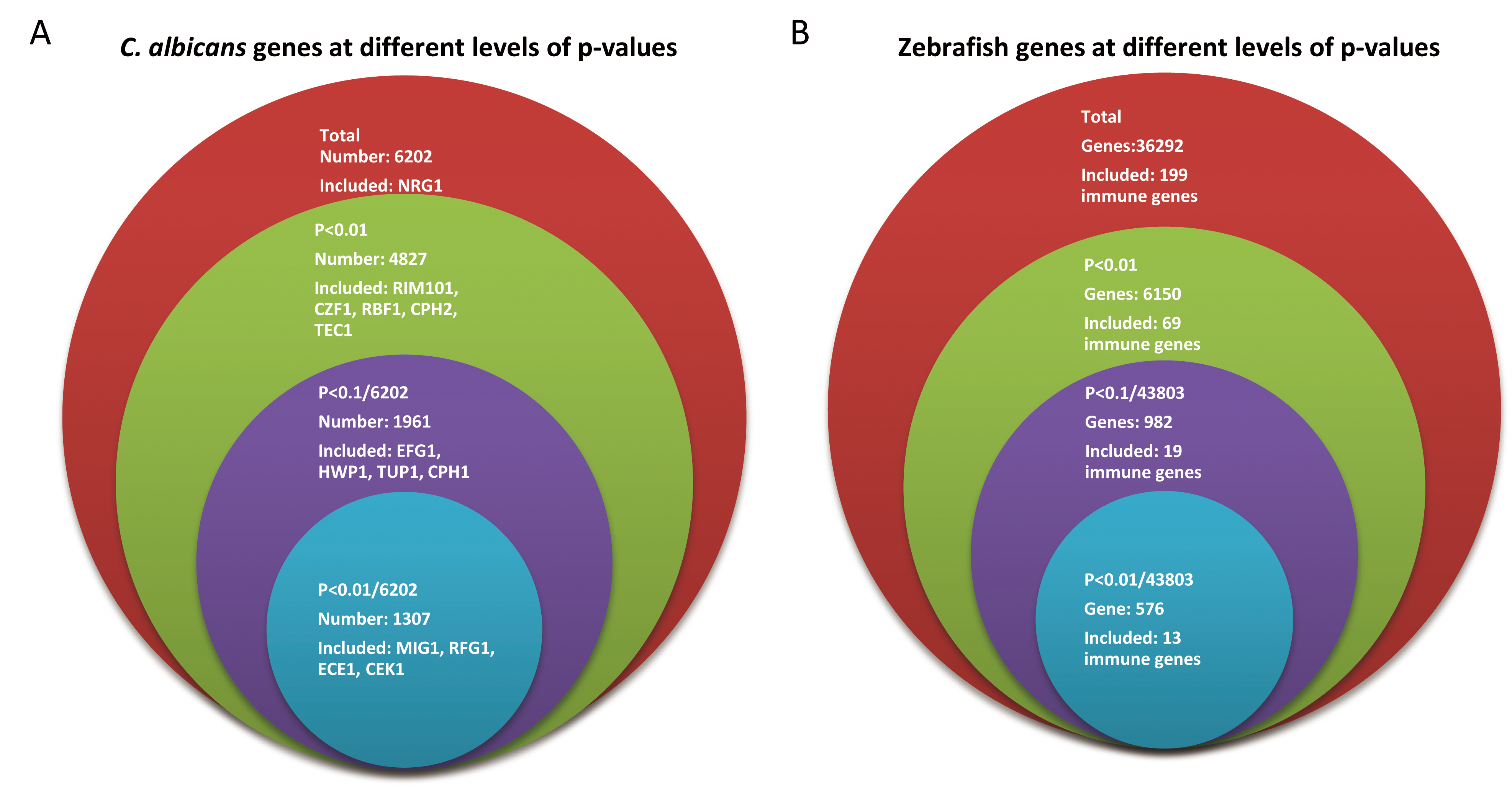

Supplement: Figure S1 — C. albicans and zebrafish genes at different p-values. (A) Venn diagram of the significant genes in C. albicans. There were a total of 4,827 significant genes with a p-value of less than 0.01, and there were 4,827 significant genes with a p-value of less than 0.1/6202 (Bonferroni correction). This study focused on 1,307 genes that fulfilled the stringent criterion of a p-value less than 0.01/6202. This list included several genes related to C. albicans hyphal formation, such as MIG1, RFG1, ECE1, and CEK1. (B) Venn diagram of the significant genes in zebrafish. There were a total of 36,292 significant genes with a p-value of less than 0.01, and there were 6,150 significant genes with a p-value of less than 0.1/43803. This study focused on 683 genes that fulfilled the same criterion used in the C. albicans analyses; namely, a p-value less than 0.01/43803 (Bonferroni correction). This gene list included key genes related to the immune response, such as IL1B, TNFA, and TNFB. (TIF) [file pone.0072483.s001.tif]

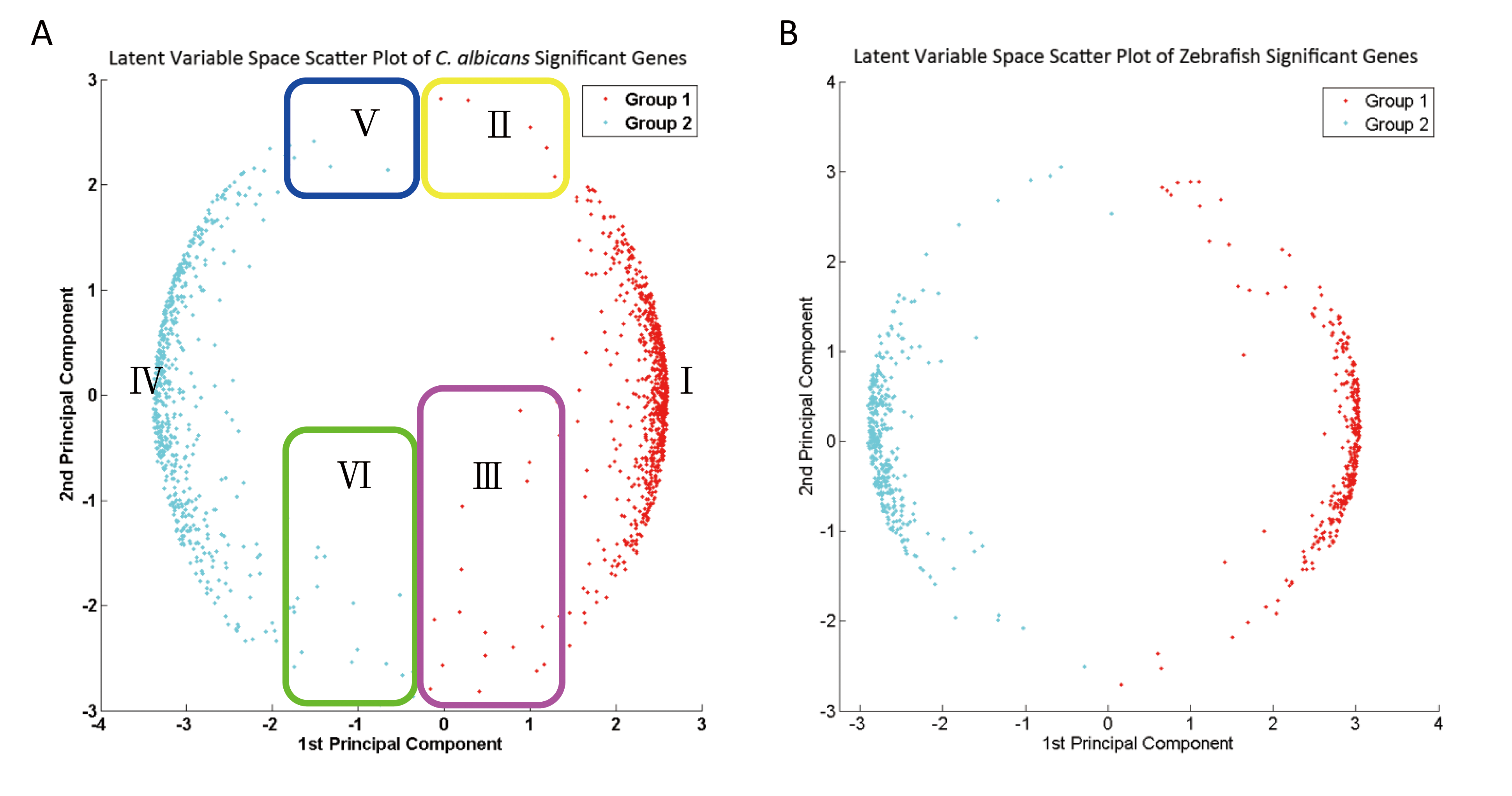

Supplement: Figure S2 — Clustering of significant genes in the PC subspace. (A) A scatter plot of the PC scores of the first two principal components demonstrating that the significant C. albicans genes could be classified into two main groups and six sub-groups. (B) A scatter plot of PC scores of the first two principal components demonstrating that the significant C. albicans genes could be classified into two main groups. (TIF) [file pone.0072483.s002.tif]
